# Renal Outcomes in Hereditary Fibrinogen A Alpha-Chain Amyloidosis

**DOI:** 10.1016/j.ekir.2026.107076

**Published:** 2026-09-08

**Authors:** Jack E. Carruthers, Muhammad U. Rauf, Hugh Goodman, Aviva Petrie, Dorota Rowczenio, Graham Taylor, Diana Canetti, Raizel Fernandes, Janet A. Gilbertson, Ana Martinez Naharro, Helen J. Lachmann, Ashutosh D. Wechalekar, Marianna Fontana, Philip N. Hawkins, Julian D. Gillmore

**Affiliations:** 1 National Amyloidosis Centre, University College London, Royal Free Hospital, London, United Kingdom; 2 Department of Haematology, Waikato Hospital, Hamilton, New Zealand

**Keywords:** AFib amyloidosis, graft survival, proteomics, rare renal disease, renal survival

## Abstract

**Introduction:**

Hereditary fibrinogen A alpha-chain (AFib) amyloidosis is characterized by progressive proteinuric chronic kidney disease (CKD) and early progression to end-stage renal disease (ESRD). A large series published in 2009 showed renal survival <5 years from diagnosis and renal transplant survival of <10 years. Since then, advances in diagnostic techniques and nephroprotective care have transformed both nephrology and amyloidosis. We evaluated contemporary diagnostic and renal outcomes in the largest cohort of patients with AFib amyloidosis to date.

**Methods:**

A total of 194 patients with confirmed AFib amyloidosis who were followed at a large national center and diagnosed between 1993 and 2025 were included. Patients were stratified by the era of diagnosis, pre- or post-2009. Diagnostic approaches, renal outcomes, transplant outcomes, and patient survival were evaluated.

**Results:**

Median age at diagnosis was 60 years with mean estimated glomerular filtration rate (eGFR) of 37 ml/min per 1.73 m^2^. Proteomics-based amyloid typing became the predominant diagnostic modality post-2009 (60.5%). 62.8% progressed to ESRD. The mean annual rate of eGFR decline was 6.7 ml/min per 1.73 m^2^ in the pre-2009 era and 4.8 ml/min per 1.73 m^2^ in the post-2009 era. Renal survival improved post-2009, particularly among patients with CKD stage IIIA or B at diagnosis. Median renal allograft survival improved from 7.7 years in the pre-2009 era to 15.5 years in the post-2009 era (*P* = 0.006) (hazard ratio [HR], 4.5 [95% confidence interval [CI], 1.2–22.6]). Overall patient survival did not differ significantly between eras (median, 12 years; *P* = 0.69, log-rank).

**Conclusions:**

Renal and allograft survival have improved since 2009, likely reflecting advances in supportive nephrology care. Isolated kidney transplantation is associated with favorable long-term outcomes. Despite improvements in renal survival, overall survival remains unchanged, underscoring the need for effective amyloid-targeted therapies.

## Graphical abstract

**Figure:**
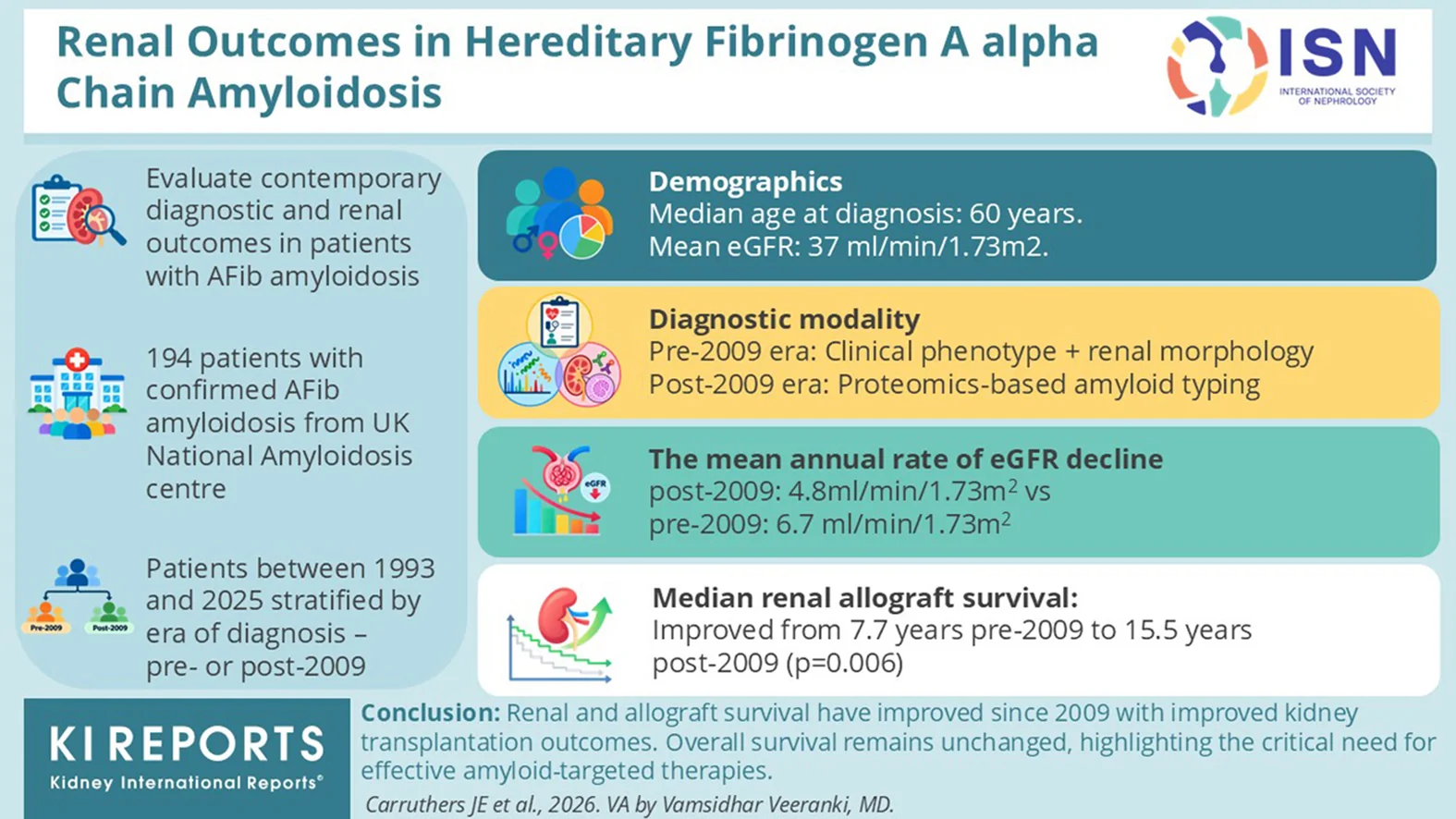

AFib amyloidosis is an autosomal dominant condition caused by mutations in the *FGA* gene resulting in production of variant fibrinogen A alpha-chain protein which misfolds into amyloid fibrils and deposits predominantly in the kidneys. Variant circulating fibrinogen A alpha-chain protein is entirely liver-derived and the wild-type protein does not form amyloid fibrils *in vivo*.^1^ Although amyloid deposits may be identified in other organs such as the spleen and occasionally the liver, the predominant clinical manifestation of AFib amyloidosis is proteinuric CKD.

AFib amyloidosis was first described by Ostertag in 1932 and the natural history and clinical course were fully characterized in 2009 by Gillmore *et al.*^2^ in a case series of 71 patients who were diagnosed and followed at the United Kingdom (UK) National Amyloidosis Centre (NAC).^3^ In this series, diagnosis was achieved mainly through a combination of family history, genetic sequencing, and characteristic morphology and immunohistochemical staining of a kidney biopsy. The natural history was characterized by progression to ESRD within median 5 years of diagnosis. Several patients underwent either isolated renal transplantation (RT) for renal replacement therapy or combined liver-kidney transplantation (LKT), the latter with curative intent. The posttransplant course was characterized by modest renal allograft survival (median <10 years) and operative complications including early mortality related to the LKT procedure.

Since 2009, there have been considerable advances in the care of patients with both renal disease generally and amyloidosis specifically. For example, identification of patients living with CKD has steadily improved across the UK with greater awareness of the need to prescribe antiproteinuric medications such as renin-angiotensin-aldosterone system inhibitors and more recently sodium glucose transporter 2 inhibitors (SGLT2i).^4^^,^^5^ Moreover, the number of renal transplants performed in the UK has increased steadily every year.^6^ In the field of AFib amyloidosis, mass spectrometry methodology has been developed to specifically determine the causative variant.^7^^,^^8^ In addition, outcomes in a range of different types of amyloidosis have improved, partly because of increased awareness and earlier diagnosis, and partly because of advances in amyloid-specific therapy.^9^, ^10^, ^11^

With these advances in mind, we sought to evaluate contemporary diagnostic practices along with renal and organ transplant outcomes in a large cohort of patients with AFib amyloidosis and reveal improving native kidney and renal allograft survival since 2009.

## Methods

### Patients

All patients with a diagnosis of AFib amyloidosis under follow-up at the NAC were included in this study, which spanned the period between 1993 and November 2025. Where informative, patients were stratified into those diagnosed in the 16 years pre-2009 and the 16 years post-2009 to facilitate comparison with our earlier published case series. The cut-off date for the pre-2009 cohort was December 31, 2008 which accounts for the slight differences in the total number of patients in this case series versus Gillmore *et al.*’s^2^ original publication in which the censor date was earlier. Patients attended NAC for their initial diagnostic work-up and were then followed at regular intervals, usually 12-monthly.

Demographic data including age at diagnosis, sex, CKD, and albuminuria stage at baseline were collected. Whether patients were taking antiproteinuric medications such as angiotensin-converting enzyme inhibitors (ACEi) or angiotensin II receptor blockers (ARB), was recorded. Patients were assessed as potentially ‘eligible’ to be prescribed sodium glucose transporter 2 inhibitors (SGLT2i), which were approved for use by the UK National Institute for health and Care Excellence in 2021–2022, if they had an eGFR >25 ml/min per 1.73 m^2^, had no obvious contraindication to SGLT2i, and were not living with a renal transplant at any point after 2022. We then recorded the number of eligible patients on SGLT2i by era. At each clinic visit, there was a comprehensive clinical assessment including vital signs, functional status, renal function, liver function, 24-hour urine protein quantification and urinary albumin-creatinine ratio measurement, electrocardiography, echocardiography, and iodine-123-labelled serum amyloid P component (^123^I-SAP) scintigraphy. A small proportion of patients underwent cardiac magnetic resonance (CMR) imaging.

Mortality date and dialysis data were obtained from the central National Health Service care records. Where informative, trends in clinical outcomes were compared between the pre-2009 cohort and the post-2009 cohort. ESRD was defined as the date of initiation of renal replacement therapy or kidney transplantation. Kidney survival was taken as the time from diagnosis of AFib amyloidosis to date of ESRD.

All patients were managed in accordance with the declaration of Helsinki, and informed patient consent and institutional review board approval from the Royal Free Hospital Ethics Committee were obtained for this study (ref: 06/Q0501/42).

### Diagnosis

The diagnosis of AFib amyloidosis was established based on the clinical phenotype in conjunction with a combination of genetic sequencing and histology. Analysis of the *FGA* gene was performed on EDTA blood samples as described previously. Briefly, exon 5 of the *FGA* gene was amplified by polymerase-chain-reaction and analyzed via automated sequencing. Amplification was carried out with Ready-To-Go tubes (VWR International Ltd., Lutterworth, UK) (primer sequences available upon request). Sequencing reactions were performed with Big Dye Terminator v 3.1 Cycle Sequencing Kit (Applied Biosystems, Foster City, CA) according to manufacturer’s instructions. Electropherograms were analyzed on the ABI 3500xl Genetic Analyser (Thermo Fisher, Waltham, MA) using Sequencing Analysis Software version 6 (Thermo Fisher, Waltham, MA).

Histology was performed on formalin-fixed, paraffin-embedded renal biopsy specimens. They were stained for amyloid with Congo red and viewed under cross polarized light by 2 independent operators. Immunohistochemical staining of amyloid deposits was performed using a panel of antibodies against serum amyloid A, kappa and lambda immunoglobulin light chains, transthyretin, and fibrinogen A alpha chain. A monoclonal sheep antihuman IgG fibrinogen A alpha chain antibody (CA1023) was used (,Calbiochem, San Diego, CA).

Proteomic analysis was performed, as described previously.^7^ Briefly, amyloid deposits from renal biopsy specimens were laser microdissected and then digested using trypsin. Proteomics analysis was undertaken on a Thermo Scientfiic Orbitrap Velos hybrid Ion Trap-Orbitrap mass spectrometer (Thermo Fisher Scientific, Bremen, Germany). Raw mass spectral data were queried through Mascot using the Swiss-Prot Human Protein database. Amyloid was identified if 2 of 3 amyloid signature proteins were present: apolipoprotein E, apolipoprotein A-IV, and serum amyloid P component. The amyloid was typed as AFib if the fibrinogen Mascot protein score was >80 with minimum 2 unique significant peptides matching the human fibrinogen peptide sequence in Swiss-Prot. After the initial database search, a modified database including the known amyloidogenic *FGA* variant peptide sequences was appended to Mascot to allow for identification of the specific AFib variant. A panel of experts in amyloid diagnostics then analyzed the proteomic data in conjunction with genetic sequencing, histology, and clinical features to definitively establish the diagnosis.

### Echocardiography and CMR imaging

Two-dimensional transthoracic echocardiograms were performed on nearly all patients presenting for diagnosis at the NAC. Echocardiograms were undertaken by experienced operators blinded to the final outcome using a GE Vivid E9 (GE Healthcare, Chicago, IL) ultrasound machine equipped with a 5S probe. Operators were blinded to the clinical details and suspected diagnosis. Echocardiogram reports were reviewed by expert cardiologists to assign diagnosis.

CMR was performed when patients were referred to the unit with diagnostic uncertainty over their amyloid diagnosis. For example, a patient may have been referred as a suspected light chain amyloidosis who later was revealed to have AFib amyloidosis. Alternatively, patients who had suspected features of cardiac amyloidosis on echocardiography that could not be explained by clinician expertise, may have undergone a CMR. CMR was performed on a 1.5-T clinical scanner (Magnetom Aera, Siemens Healthcare, Erlangen, Germany). Within a conventional clinical scan, late gadolinium enhancement imaging was acquired and T1 measurement was performed using the modified looklocker inversion recovery sequence. After a bolus of gadoterate meglumine (0.1 mmol/kg, gadolinium-DOTA, Dotarem, Guerbet S.A., France) and late gadolinium enhancement imaging, T1 mapping was repeated 15 minutes postcontrast using the same slice locations, to produce automated inline extracellular volume mapping reconstruction. All CMR imaging analysis was performed blinded to clinical details and diagnosis.^12^

### SAP Scintigraphy

Whole-body anterior and posterior scintigraphic imaging using an Elscint Superhelix gamma camera was undertaken 6 or 24 hours after administration of ^123^I-labelled SAP, as previously described.^13^ Some patients underwent serial ^123^I-SAP scintigraphy for quantitation of visceral amyloid burden over time.

### Statistical Analyses

Statistical analysis was performed using GraphPad Prism (version 11.0.0; GraphPad, La Jolla, CA) and Microsoft Excel. Kaplan-Meier survival analyses were undertaken to establish renal and patient survival from date of diagnosis of AFib amyloidosis to event. Multivariable Cox regression analyses were undertaken using clinically relevant variables from univariable analyses to identify independent predictors of renal survival and overall survival. To account for the possibility of follow-up time asymmetry biasing renal survival between the 2 groups, a separate survival analysis for time from diagnosis to ESRD was undertaken using a truncated follow-up structure. Given the maximum follow-up time for patients in the post-2009 group was 16 years, we truncated follow-up for patients in the pre-2009 era at 16 years after diagnosis and repeated Kaplan-Meier analyses in this adapted cohort. Censor date was November 1, 2025. Rate of eGFR decline was calculated for all patients with baseline eGFR >20 ml/min per 1.73 m^2^ and with at least 3 documented eGFR readings over the course of their follow-up and subsequently stratified by era of diagnosis. Death censored renal allograft survival was calculated by Kaplan-Meier survival analyses with renal allograft survival stratified by transplant date (pre- and post-2009) and compared by log-rank test. Patients with incomplete longitudinal data were excluded from survival analyses. HRs with 95% CI were generated for event rates including renal survival stratified by National Kidney Foundation-Kidney Disease Outcome Quality Initiative CKD stage, renal allograft survival, and overall patient survival. Quantitative variables were compared using *t* test or chi square where appropriate.

## Results

### Demographics

There were 194 patients with AFib amyloidosis included in the cohort; 75 were diagnosed pre-2009 and 119 were diagnosed post-2009. A summary of the baseline patient characteristics for the whole cohort and the separate pre-2009 and post-2009 cohorts is shown in Table 1. The median (range) age at diagnosis was 60 (39–84) years and did not change between the 2 eras (*P* = 0.07). There was a slightly higher proportion of males (55.6%) than females in the cohort. The proportion of patients in each CKD stage at the time of diagnosis is shown in Table 1. Notably, nearly one-third (30.4%) of the cohort were in CKD stage IV by the time of diagnosis (CKD stage IV, 19/75 [25.3%] vs. 40/119 [33.6%], pre- and post-2009, respectively). Most patients had A3 proteinuria at the time of diagnosis.Mean (range) urinary protein quantification at diagnosis was 4.6 (0.1–19.0) g/day and mean urinary albumin-creatinine ratio was 384 (8–1577) mg/mmol. Of note, 15 (7.7%) patients were dialysis-dependent at the time of diagnosis of AFib amyloidosis and the remainder (92.2%) were dialysis independent. Mean (range) eGFR at diagnosis among those who were dialysis independent was 37 (<15–>90) ml/min per 1.73 m^2^.

**Table 1:** Cohort characteristics and parameters at diagnosis

Characteristics	Total	Pre-2009	Post-2009	*P*-value
Patients; *N*	194	75	119	
Males; *n* (%)	108 (55.6)	41 (54.7)	67 (56.3)	0.82
Age; median (range)	60 (39–84)	59 (39–80)	61 (43–84)	0.07
Presentation				
eGFR; mean (ml/min per 1.73 m^2^)	37	42	34	0.04
Proteinuria; mean (g/d)	4.6	4.7	4.6	0.84
CKD stage; *n* (%)				
I	12 (6.2)	6 (8.0)	6 (5.0)	
II	23 (11.9)	10 (13.3)	13 (10.9)	
IIIA	13 (6.7)	6 (8.0)	7 (5.9)	
IIIB	31 (15.9)	11 (14.7)	20 (16.8)	
IV	59 (30.4)	19 (25.3)	40 (33.6)	
V	34 (17.5)	9 (12.0)	25 (21.0)	
ESRD/RRT-requiring	15 (7.7)	12 (16.0)	3 (2.5)	
No data	7 (3.6)	1 (0.5)	6 (5.0)	
Albuminuria stage; *n* (%)				
A1	0 (0)	0 (0)	0 (0)	
A2	9 (4.6)	5 (6.7)	4 (3.4)	
A3	163 (84.0)	56 (74.7)	107 (90.0)	
Medications; *n* (%)				
ACEi/ARB	91 (47.0)	25 (33.3)	66 (55.5)	
Eligible for SGLT2i	39 (20.1)	1 (1.3)	38 (19.6)	
SGLT2i; *n* (% eligible)	9 (23.1)	0 (0.0)	9 (23.6)	
Missing	23 (11.9)	18 (24.0)	5 (4.2)	
Diagnosis				
IHC; *n* (%)	68 (35.1)	35 (46.7)	33 (27.7)	
Proteomics; *n* (%)	96 (49.5)	24 (32)	72 (60.5)	
Genetics; *n* (%)	30 (15.4)	16 (21.3)	14 (11.8)	

ACEi, angiotensin converting enzyme inhibitor; ARB, angiotensin receptor blocker; eGFR, estimated glomerular filtration rate; IHC, immunohistochemistry; KDOQI, Kidney Disease Outcome Quality Initiative; SGLT2i, sodium glucose transporter 2 inhibitor; uACR, urinary albumin-creatinine ratio.

CKD and albuminuria stages correspond to eGFR and uACR KDOQI cut-offs. Where appropriate, outcomes pre- and post-2009 were compared using unpaired *t* test or chi square with *P* < 0.05 being deemed significant.

A greater proportion of patients in the post-2009 era were treated with ACEi or ARB (25/75 [33.3%] pre-2009 vs. 66/119 [55.5%] post-2009), although there were more missing data in the pre-2009 cohort (18/75 [24.0%] pre-2009 vs. 5/119 [4.2%] post-2009). Only 1 patient was considered eligible for SGLT2i prescription in the pre-2009 cohort who was not prescribed SGLT2i. Out of 38, 9 (23.7%) eligible patients were prescribed SGLT2i in the post-2009 cohort.

### Diagnosis

In the pre-2009 era, the diagnosis was established based on the clinical phenotype in conjunction with renal morphology and genetic sequencing in all patients. Immunohistochemistry was diagnostic of AFib-type amyloid in 35/75 (46.7%) patients. Post-2009, the predominant method of diagnosis was proteomic analysis, which was performed in 72/119 (60.5%) patients, coupled with clinical phenotypic findings, renal morphology, and genetic sequencing. Available biopsy specimens from the pre-2009 cohort were processed retrospectively for proteomic analysis which confirmed AFib-type amyloid coupled with the associated *FGA* gene variant in 24/24 (100%) cases.

All patients underwent genomic sequencing of the *FGA* gene. Most patients (187/194 [96.4%]) were heterozygous for the previously described single base substitution that alters codon 526 of the mature protein from glutamic acid to valine (p.Glu545Val; formerly E526V). Of the remaining 7 patients, 3 (1.5 %) carried the p.Glu559Val (formerly E540V) variant, 2 (1.0 %) had the p.Arg573Leu (R554L) variant, and 1 each had the p.Pro571His (P552H) and p.Gly574Phe (G555F) variants.

### Natural History and Renal Progression

The mean rate of eGFR loss among patients with an eGFR >20 ml/min per 1.73 m^2^ at diagnosis was 5.3 ml/min per 1.73 m^2^ per year (6.7 ml/min per 1.73 m^2^ per year in the pre-2009 era vs. 4.8 ml/min per 1.73 m^2^ per year in the post-2009 era). Progression to ESRD requiring renal replacement therapy occurred in 122/194 (62.8%) cases. Median (range) age at initiation of renal replacement therapy was 61 (36–84) years. Comparison of the time from diagnosis to ESRD between the pre-2009 and post-2009 cohorts stratified by CKD stage at diagnosis suggested the possibility of a slower rate of GFR loss in recent times, especially in CKD stage III. Renal survival in patients with CKD stage I or II at diagnosis was a median (95% CI) of 6.6 (2.7–14.8) years in the pre-2009 era and was not reached in the post-2009 era with an HR (95% CI) for ESRD of 1.9 (0.8–5.3) (*P* = 0.13; log-rank). Among those with CKD stage IIIA or B at diagnosis, median time to ESRD was 2.1 (1.0–4.0) years in the pre-2009 era versus 6.8 (3.2-not reached) years in the post-2009 era with an HR (95% CI) for reaching ESRD in the pre- versus post-2009 era of 3.4 (1.5–7.5) (*P* = 0.0007; log-rank test). For patients with CKD stage IV at diagnosis, median renal survival was 1.5 (0.9–1.6) years in the pre-2009 era and 1.6 (1.2–1.9) years in the post-2009 era with an HR (95% CI) for ESRD pre- versus post-2009 of 1.4 (0.7–2.7) (*P* = 0.35; log-rank test) (Figure 1). A multivariable Cox regression for predicting time from diagnosis to ESRD identified era of diagnosis as the strongest predictor of renal survival (HR, 0.44 [95% CI, 0.28–0.70] post-2009; *P* = 0.018), followed by age at diagnosis (HR, 0.95 [95% CI, 0.92-0.98]; *P* = 0.0003), eGFR at baseline (HR, 0.97 [95% CI, 0.96-0.99]; *P* < 0.0001) and baseline 24 hour proteinuria (HR, 1.08 [95% CI, 1.00-1.16]; *P* = 0.04) when adjusted for sex and ACEi or ARB therapy (Table 2).

**Figure 1:**
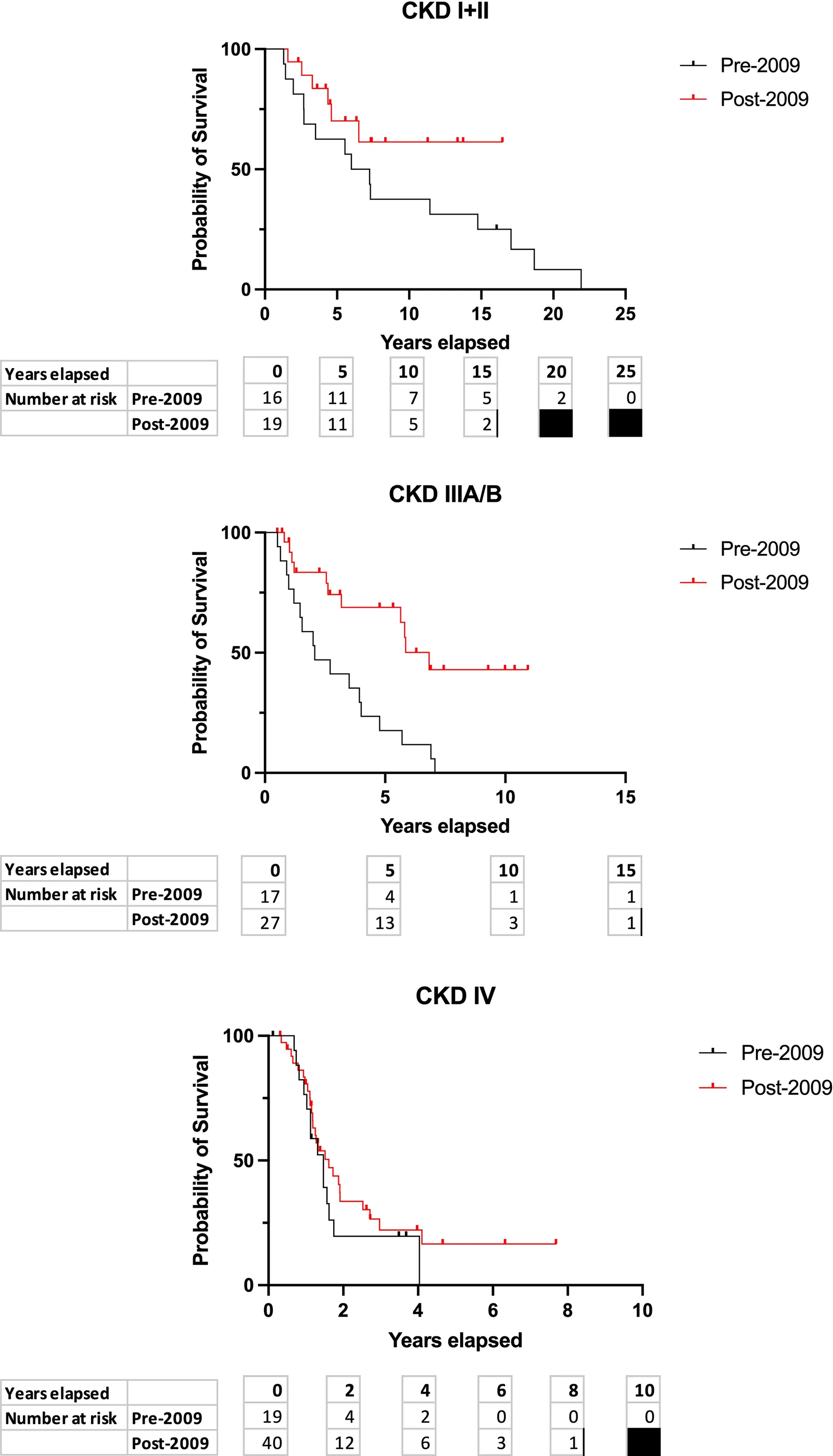
Kaplan-Meier analysis of renal survival from diagnosis to date of RRT or censor by CKD stage stratified by the era (pre- and post-2009). Patients with CKD V (eGFR < 15) at diagnosis were excluded from subgroup analysis. For CKD I+II, *P* = 0.13; for CKD IIIA or B, *P* = 0.0007; for CKD IV, *P* = 0.35, all by log-rank test. CKD, chronic kidney disease; eGFR, estimated glomerular filtration rate; RRT, renal replacement therapy.

**Table 2:** Multivariable Cox proportional hazards model for renal and overall survival

Variables	HR (95% CI)	*P*-value
Renal survival		
Age	0.95 (0.92–0.98)	0.0003
Sex	0.99 (0.65–1.52)	0.99
eGFR	0.97 (0.96–0.99)	< 0.0001
24 h proteinuria	1.08 (1.00–1.16)	0.04
Era of diagnosis	0.44 (0.28–0.69)	0.0004
Medication		
None	Reference	
ACEi/ARB	0.88 (0.57–1.36)	0.56
Overall survival		
Age	1.04 (1.00–1.08)	0.03
Sex	0.91 (0.53–1.55)	0.73
eGFR	0.99 (0.98–1.00)	0.34
24 h proteinuria	0.99 (0.92–1.07)	0.85
Era of diagnosis	0.72 (0.39–1.31)	0.29
ESRD		
Yes	Reference	
No	0.80 (0.36–1.74)	0.59
Renal transplant		
No	Reference	
Yes	0.54 (0.29–0.99)	0.05
Hypertension	1.09 (0.66–1.81)	0.73
IHD	1.00 (0.54–1.81)	0.99

ACEi, angiotensin-converting enzyme inhibitor; ARB, angiotensin II receptor blocker; CI, confidence interval; eGFR, estimated glomerular filtration rate; ESRD, end-stage renal disease; h, hour(s); HR, hazard ratio; IHD, ischemic heart disease.

The time from diagnosis to ESRD was assessed in a truncated follow-up model with a fixed follow-up time of 16 years to account for any follow-up time asymmetry. Of note, 3 patients with CKD stage I or II who reached ESRD after 16 years in the pre-2009 group were excluded. No patients in the CKD stage IIIA or B and IV cohorts reached ESRD after 16 years of follow-up. HR for reaching ESRD for each CKD stage are presented in Table 3 but this analysis supports the conclusions of increased renal survival in the post-2009 era established in the unadapted Kaplan-Meier analyses. Indeed, the median time for reaching ESRD in patients with CKD stage I or II was significantly different pre- versus post-2009 (pre-2009 4.5 [1.4–7.3] vs. post-2009 not reached; HR 3.6 [95% CI, 1.4–9.5]; *P* = 0.02).

**Table 3:** Renal survival in a truncated analysis with maximum follow-up time of 16 years

CKD stage	Patients excluded (*n*)	Renal survival; median (95% CI)		HR (95% CI)	*P*-value (log-rank)
		Pre-2009	Post-2009		
CKD I/II	3	4.5 (1.4–7.3)	Undefined	3.6 (1.4–9.5)	0.02
CKD IIIA/B	0	2.1 (1.0–4.0)	6.8 (3.2–undefined)	3.4 (1.5–7.5)	0.0007
CKD IV	0	1.5 (0.9–1.6)	1.6 (1.2–1.9)	1.4 (0.7–2.7)	0.35

CI, confidence interval; CKD, chronic kidney disease; HR, hazard ratio.

Patients in the pre-2009 group were truncated at 16 years follow-up time and Kaplan-Meier survival analysis was repeated in this adapted cohort versus post-2009 era. *P* < 0.05 is statistically significant.

Among 122 patients who reached ESRD, 71 started hemodialysis, 37 started peritoneal dialysis and 14 underwent preemptive RT. Sixteen patients (43.3%) who commenced peritoneal dialysis later converted to hemodialysis after a median (range) time on peritoneal dialysis of 1.4 (0.5–3.4) years. Out of 194, 48 (24.7%) received an isolated RT and 13 (6.7%) underwent combined LKT equating to receipt of a kidney allograft in exactly 61/122 (50%) of those with ESRD.

### Extra-renal Amyloid

^123^I-SAP scintigraphy was performed on at least 1 occasion in 192/194 (99.0%) patients from the cohort. Splenic amyloid was identified by SAP scintigraphy in 155/192 (80.7%), adrenal amyloid in 33/192 (17.2%), and hepatic amyloid in 12/192 (6.3%) of scanned patients. Out of 12, 3 patients with hepatic amyloid had a progressive rise in alkaline phosphatase and gamma-glutaryltransferase attributable to their hepatic amyloid. Despite median follow up from diagnosis of 9.8 years among the 12 patients with hepatic amyloid, no such patient developed failure of liver synthetic function. Out of 12 patients, 1 with hepatic amyloid underwent a combined LKT, the purpose of which was to prevent recurrence of amyloid in the renal allograft.

Echocardiography showed no evidence of cardiac amyloidosis in most patients although 12/194 (6.2%) were thought to have certain features that were consistent with cardiac amyloidosis such as thickened heart walls and abnormal strain imaging at their referring local centers. More detailed evaluation of these 12 patients on echocardiography at NAC, revealed that the echocardiographic features were not typical of cardiac amyloidosis and were attributable to alternative causes. All 12 patients had a long history of hypertension and 10/12 were receiving hemodialysis and had evidence of volume overload, a well-established cause of echocardiographic features that can mimic cardiac amyloidosis in the absence of specialist evaluation. Among 12 additional patients who underwent CMR imaging, an imaging modality which can distinguish uremic cardiomyopathy from cardiac amyloidosis, there was no evidence of cardiac amyloidosis in any case.

### Transplant Outcomes

Overall, 13 patients underwent combined LKT between 1996 and 2023, 9 patients in the pre-2009 cohort and 4 in the post-2009 cohort suggesting that LKT has become less favored over time. Out of 9, 3 (33.3%) patients in the pre-2009 cohort died because of procedure-related complications within 1 year of LKT, 1 died from necrotizing pancreatitis and the others from perioperative complications. One patient died with deteriorating hepatic function in the context of liver allograft biliary strictures 16 years after LKT. All 4 patients who underwent LKT after 2009 were alive at the time of Censor. At Censor, a median of 13.6 years after LKT, 6 patients had died including 2 with both allografts functioning at the time of death. Of note, 7 patients were alive, 5 with their original 2 allografts still functioning, 2 with a second kidney transplant because of failure of the original renal allograft from transplant glomerulopathy and calcineurin inhibitor toxicity respectively, and 1 was back on dialysis.

Overall, 48 patients underwent isolated RT between 1996 and 2023 including 11 in the pre-2009 era and 37 in the post-2009 era. Amyloid recurrence leading to RT failure occurred in 8 patients (4/11 pre-2009 and 4/37 post-2009); 1 patient in the pre-2009 cohort lost 2 renal allografts after 5.8 and 7.0 years because of amyloid recurrence in each allograft. In addition, there were 5/37 patients in the post-2009 group judged to have amyloid recurrence but with functional transplants at the time of Censor. The proportion of transplants that showed recurrence was 5/12 (41.6%) in the pre-2009 era and 9/37 (24.3%) in the post-2009 era. Out of 12, 5 renal allografts failed from the pre-2009 cohort and 5/37 failed from the post-2009 cohort, with 1 failing because of operative factors 2 weeks after implantation. Median renal allograft survival by Kaplan-Meier analysis was 7.7 (5.8 to undefined) years in the pre-2009 cohort and 15.5 (10.0 to undefined) years in the post-2009 cohort (*P* = 0.006, log-rank test) (Figure 2). The rate of renal allograft failure in the pre-2009 cohort was significantly higher than post-2009 cohort with an HR of 4.5 (95% CI, 1.2 to 22.6).

**Figure 2:**
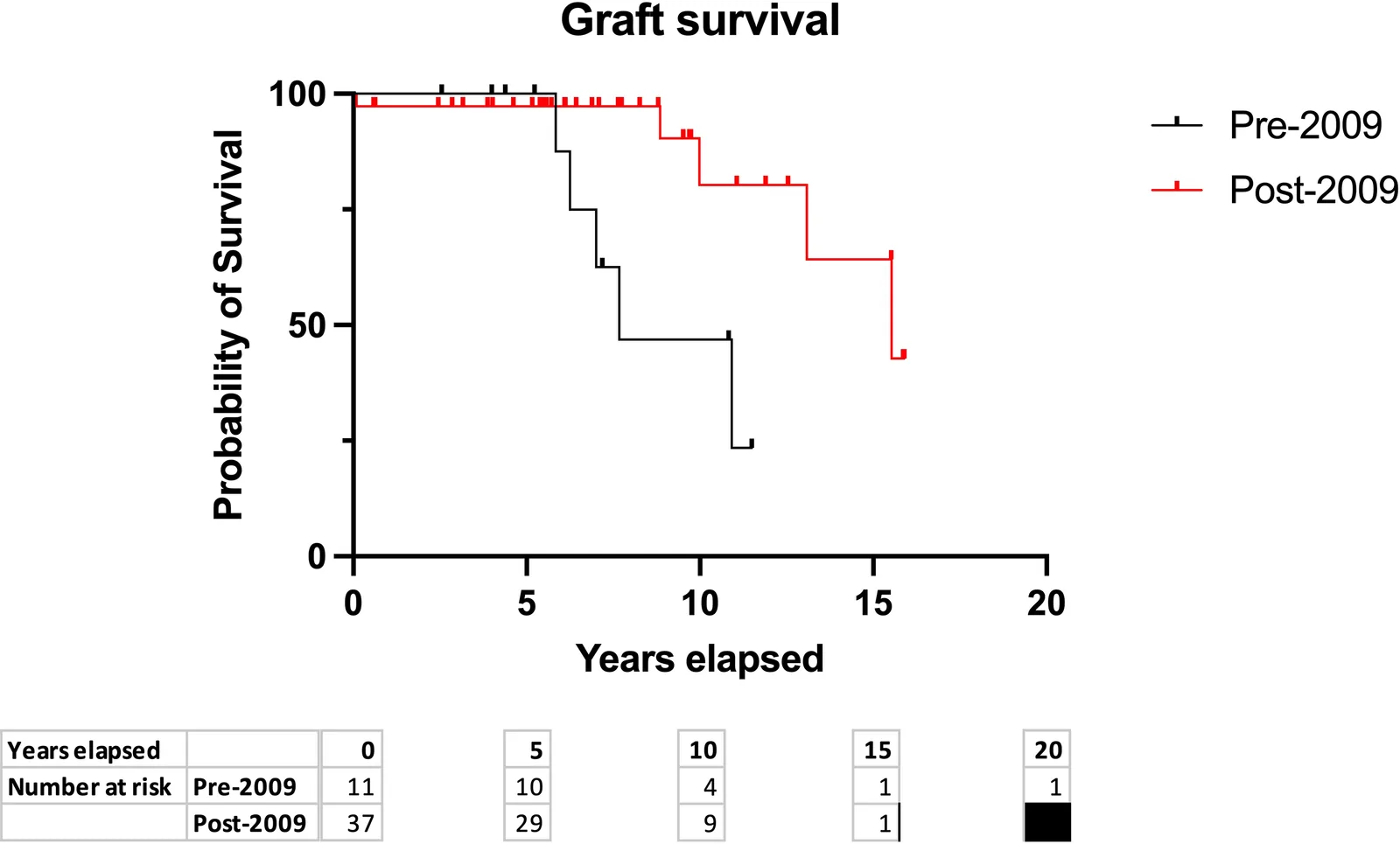
Kaplan-Meier analysis of death censored renal allograft survival from date of transplant to date of transplant failure or Censor stratified by the era (pre- and post-2009). *P* < 0.005, by log-rank test.

### Cardiovascular Disease

CKD is known to be an independent risk factor for cardiovascular disease and we assessed indices of cardiovascular health in the whole AFib amyloidosis cohort. Most patients (103/194 [53.1%]) presented with hypertension at the time of diagnosis and 39/194 (20.1%) had evidence of ischemic heart disease, confirmed by angiography, during the course of their lifetime including 16 patients who underwent percutaneous coronary intervention and 6 who had coronary artery bypass grafts. Cerebrovascular disease was also evident with at least 9 cerebrovascular accidents or transient ischemic attacks recorded in the cohort. Of note, 2 patients underwent carotid endarterectomies and peripheral vascular disease was documented in 3 patients.

### Patient Survival

There was no significant difference in patient survival in the pre- and post-2009 cohorts; 50/75 (66.7%) of the pre-2009 cohort had died at the time of Censor at a median (range) age of 72 (56–88) years and 32/119 (29.4%) of the post-2009 cohort had died at the time of Censor at a median (range) age of 69 (48–85) years (*P* = 0.07). By Kaplan-Meier analysis, median (95% CI) survival from diagnosis was 12.7 (10.0–19.1) years and 12.2 (9.85–undefined) years in the pre- and post-2009 cohorts respectively (*P* = 0.69, log-rank test) (Figure 3). Cox regression revealed that age at diagnosis was a significant predictor of death, with an HR of 1.04 (95% CI, 1.00–1.07; *P* = 0.03) when adjusted for sex, 24-hour urine proteinuria, eGFR, diagnostic era, presence of ESRD, presence of a renal transplant, hypertension, and ischemic heart disease. Presence of a renal transplant conferred an HR of 0.54 (95% CI, 0.29–0.99; *P* = 0.05) on the risk of death (Table 2).

**Figure 3:**
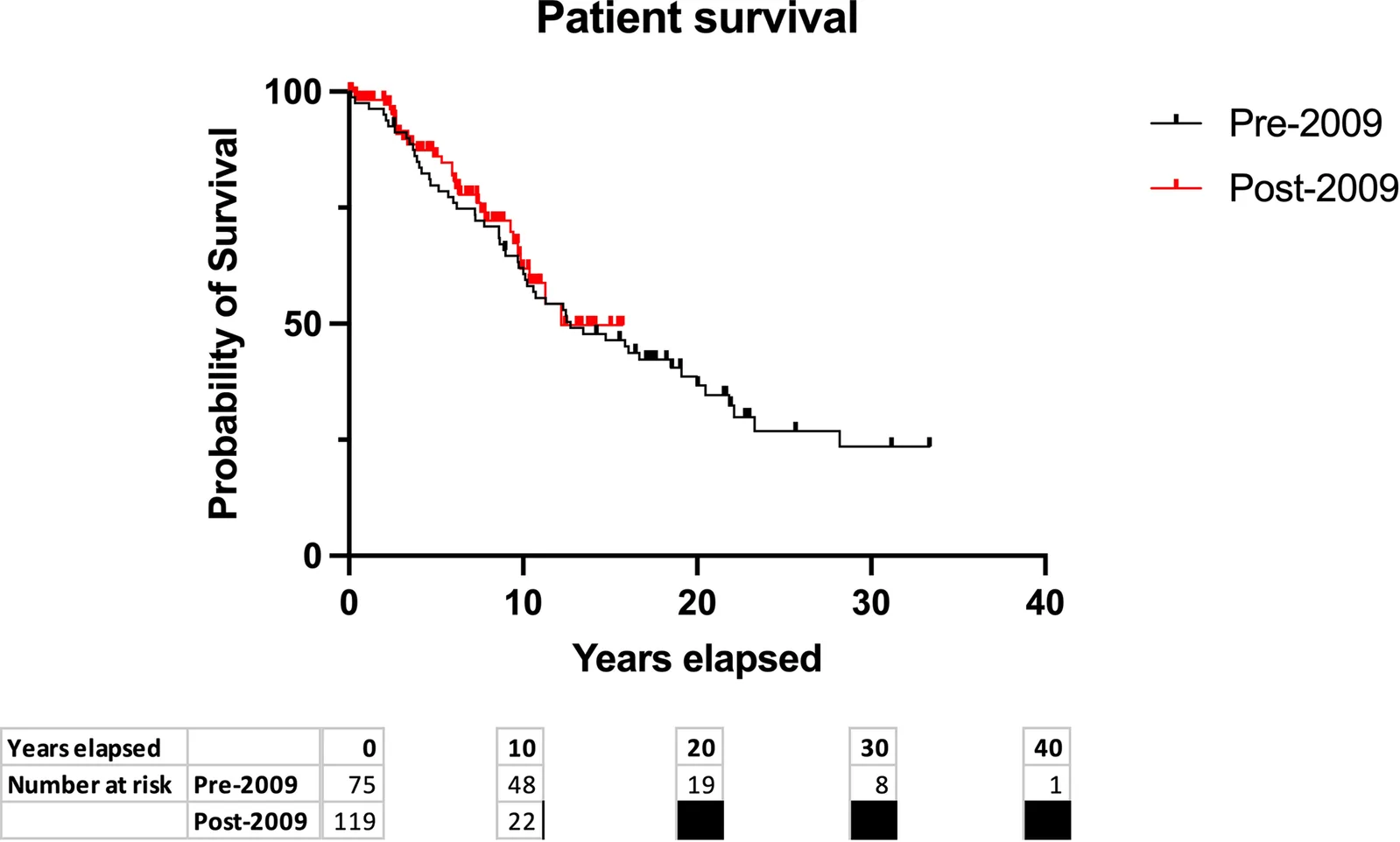
Kaplan-Meier analysis of overall patient survival from diagnosis to death or Censor stratified by the era (pre- and post-2009). *P* = 0.69 by log-rank test.

## Discussion

This study represents, to our knowledge, the largest reported series of patients with AFib amyloidosis and the 1 with the most prolonged longitudinal follow-up, substantially extending our original 2009 report. The size and duration of follow-up provide a unique opportunity to define contemporary disease trajectory, evolving diagnostic pathways, and long-term renal and transplant outcomes in this rare disorder.

The diagnostic landscape has evolved considerably over the past decade. Mass spectrometry-based proteomics has now become central to amyloid typing generally, and in AFib amyloidosis because it specifically overcomes the recognized limitations of immunohistochemistry, not only in enabling precise identification of the amyloid fibril protein but also the specific pathogenic fibrinogen variant.^7^ When combined with sequencing of the *FGA* gene, this approach provides a standardized and highly robust diagnostic pathway for AFib amyloidosis, particularly given an ever-expanding spectrum of amyloidogenic protein variants. Indeed, at least 6 novel amyloidogenic variants in *FGA* have been described since 2009 including 4 single base substitutions and 2 frameshift mutations, leading to a total of 16 published variants in *FGA* (www.amyloidosismutations.com/). Of note, 2 of those variants were first described in patients assessed at NAC.^14^, ^15^, ^16^ The increase in the availability of both sequencing and proteomics as well as a change in renal diagnostic practices may explain why the mean eGFR at baseline was apparently lower in patients presenting post-2009 than pre-2009. With more tools available for diagnosis and more extensive use of renal biopsy to diagnose causes of proteinuric CKD including among patients with more advanced CKD, a greater proportion of patients with CKD stage III–V at baseline were successfully diagnosed post-2009 than pre-2009. It is possible that before 2009, a higher proportion of patients with more advanced CKD reached ESRD without ever establishing a diagnosis of AFib amyloidosis and so were never referred to NAC, whereas only those who had a biopsy, generally at earlier stage proteinuric CKD, were ever referred and diagnosed.

A key finding of the present study is the suggestion of improving renal outcomes over time, with a slower rate of decline in kidney function in patients diagnosed in the contemporary era, especially in patients with CKD stage IIIA or B at diagnosis. Cox regression analysis revealed that age at diagnosis, baseline eGFR, baseline urinary protein quantification, and era of diagnosis were the strongest predictors of reaching ESRD. It is unclear what is driving the impact of era of diagnosis on renal survival. Given the absence of novel anti-amyloid therapy for AFib amyloidosis, it is possible that advances in general nephroprotective care, including optimization of renin-angiotensin-aldosterone system] blockade, improved access to nephrology specialists and improved blood pressure control since-2009 explains this phenomenon. Since 2021–2022, SGLT2i have been approved by National Institute for health and Care Excellence for proteinuric CKD from any cause and it remains to be seen whether widespread adoption of this class of medications will have benefit in patients with AFib amyloidosis.^4^^,^^5^ These hypotheses are supported by the observation that patients with CKD stage IIIA or B at diagnosis, an eGFR that frequently precipitates the first referral to secondary nephrology care and a patient cohort that has been shown to derive the greatest benefit from nephroprotective drugs like ACEi and ARB, showed the largest increase in renal survival post 2009.^17^ Despite this, Cox regression analyses did not reveal a significant impact of ACEi and ARB prescription on the HR of reaching ESRD, when adjusted for numerous other possible confounders, possibly because of the high proportion of missing prescription data in the pre-2009 cohort. Additionally, follow-up time asymmetry may have biased renal survival data, although we have attempted to account for this by undertaking a truncated analysis with symmetrical follow-up time that reproduced our broader conclusions. Despite these limitations, our data suggest that timely access to nephrology services for patients with AFib amyloidosis may be an important determinant of disease-related outcomes.

The data indicate that renal transplant allograft survival has improved substantially in the modern era, which likely reflects, as in the pretransplant population, improved overall care in transplant nephrology. This observation is tempered by noting the shorter follow-up time in the post-2009 cohort which may have introduced bias, highlighted by the wide CI when comparing outcomes between the pre- and post-2009 cohorts.

An intriguing but unexplained possibility is that allograft failure from recurrent amyloid deposition has diminished in recent times, possibly contributing to increased graft survival. Although we do not reliably capture the reasons for undertaking a kidney transplant biopsy, as far as we are aware all biopsies in the cohort were undertaken because of allograft dysfunction rather than as “per protocol” biopsies. Assuming the reduction in amyloid recurrence is true despite the inherent limitations of this retrospective analysis, it remains unexplained and warrants further study. Although the causative *FGA* mutation may influence amyloid recurrence risk as shown in a recent French series where 2/9 renal transplants in E526V carriers showed amyloid recurrence versus 5/6 renal transplants in non-E526V carriers, only 1 non-E526V carrier was transplanted in our cohort and showed robust graft survival such that this is not the explanation for differences in our own cohort.^18^ The extended graft survival in the post-2009 cohort has important clinical implications. Historically, combined LKT was suggested as a potentially curative strategy by eliminating hepatic production of variant fibrinogen.^19^ Although LKT does indeed appear to have eliminated any risk of recurrent amyloid in the renal allograft, it continues to be associated with significant procedural risk, which coupled with increasingly favorable outcomes after isolated RT, may have led to the declining trend in the use of LKT in the post-2009 cohort. Despite the small numbers, our data suggest that isolated RT is likely to be appropriate for most patients with ESRD because of AFib amyloidosis, although LKT may still be a valid option for younger, fitter patients.

Cardiovascular morbidity was common within the cohort, consistent with the high burden of vascular disease in CKD generally.^20^ However, despite previous reports suggesting possible cardiac involvement in AFib amyloidosis, we found no convincing evidence of clinically significant cardiac amyloid infiltration, with imaging abnormalities attributable to hypertension, ischemic heart disease, or volume-related cardiomyopathy.^21^, ^22^, ^23^ These findings reinforce that despite presence of extra-renal amyloid deposits, particularly in the spleen, AFib amyloidosis is a predominantly renal-limited clinical amyloidosis syndrome.

Despite encouraging improvements in renal and transplant outcomes, overall survival has not significantly changed, likely reflecting competing risks from renal disease *per se*, cardiovascular disease and the absence of disease-modifying therapies targeting the underlying amyloidogenic process. Importantly, our interpretation of death data is limited by the fact that our database does not capture cause of death. The strongest modifiable protective factor against death was the presence of a renal transplant, highlighting again, the importance of access to nephrological care for patients with AFib amyloidosis. The lessons from other amyloidoses suggest that there is reason to anticipate amyloid-directed therapies that may alter risk of death. These may include agents that promote clearance of existing amyloid deposits,^24^ or bind to variant fibrinogen in the circulation and facilitate its removal, or suppress production of the pathogenic protein, for example through allele-specific gene silencing or gene-editing strategies targeting the mutant *FGA* allele. Such approaches, analogous to therapeutic advances seen in other forms of systemic amyloidosis,^25^ hold the potential to slow progression or even prevent amyloidosis, extend renal survival in those with established amyloidosis, and eliminate any risk of amyloid recurrence after renal transplantation.

We acknowledge several limitations in this manuscript, mostly related to its retrospective nature. These include lack of data on cause of death or reason for native renal or renal transplant biopsy and lack of detailed data on date of commencement of renoprotective therapies or details of peritransplant immunosuppressive regimens. A comparison of outcomes by *FGA* mutation was not undertaken because of the small number of patients with non-E526V AFib amyloidosis.

In conclusion, this large, contemporary series shows that outcomes in AFib amyloidosis are evolving, with signals of slower renal decline and improved renal allograft survival in the modern era. These gains likely reflect advances in supportive renal care generally. Improving allograft outcomes with isolated RT challenge the historical paradigm favoring combined LKT and potentially support a more conservative surgical approach. However, the absence of effective amyloid-targeted therapies remains the principal limitation in care, underscoring the need for continued translational efforts to develop treatments capable of modifying or preventing this disease.

## Disclosure

All the authors declared no competing interests.
